# Species sensitivities to a global pollutant: A meta‐analysis on acoustic signals in response to anthropogenic noise

**DOI:** 10.1111/gcb.15428

**Published:** 2020-12-01

**Authors:** Hansjoerg P. Kunc, Rouven Schmidt

**Affiliations:** ^1^ School of Biological Sciences Queen's University Belfast Belfast UK

**Keywords:** animal behaviour, anthrophony, conservation, global change, human‐induced environmental change, pollution, soundscape

## Abstract

Anthropogenically driven environmental changes affect our planet at an unprecedented rate. Among these changes are those in the acoustic environment caused by anthropogenic noise, which can affect both animals and humans. In many species, acoustic communication plays a crucial role to maintain social relationships by exchanging information via acoustic signals. However, how species relying on acoustic communication differ in their adjustments to anthropogenic noise is little understood. Yet, this is crucial because protecting species effectively depends on our capability to predict how species differ in their response to human‐induced environmental changes. Using a phylogenetically controlled meta‐analysis, we quantified differences in adjustments of acoustic signals to anthropogenic noise among species. The effect sizes included in the analysis were obtained from noise exposure experiments, as only carefully controlled experiments allow to establish cause‐and‐effect relationships. We found that animals changed acoustic signals when exposed to noise, but the magnitude and the direction of adjustments differed among species. Given the importance of communication in the animal kingdom, these adjustments can affect social relationships in many species. The diversity of responses among species highlights the necessity to assess the effect of environmental stressors not only for a few species, because an effect may be positive in one species but negative in another depending on the species’ biology. Thus, an effective conservation approach to protect different species is to preserve natural soundscapes of ecosystems to which species have adapted to by reducing or mitigating the emission of anthropogenic noise into the environment.

## Graphical abstract

Using a phylogenetically controlled meta‐analysis, we quantified differences in adjustments of acoustic signals to anthropogenic noise. We found that animals changed acoustic signals when experimentally exposed to noise, but the magnitude and the direction of adjustments differed among species. Thus, an effective conservation approach to protect different species is to preserve natural soundscapes of ecosystems to which species have adapted to by reducing or mitigating the emission of anthropogenic noise into the environment.

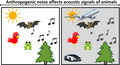

## 1 INTRODUCTION

Anthropogenically driven environmental changes affect our planet at an unparalleled scale and are considered to be a key threat to biodiversity and the function of ecosystems (Stenseth et al., 2002; Walther et al., 2002). In addition to climate change, species invasions, habitat loss, and fragmentation (Butchart et al., 2010; Dukes & Mooney, 1999; Foley et al., 2005; Thomas et al., 2004), other factors also play a critical role in eroding biodiversity such as changes in the acoustic environment caused by anthropogenic noise. In both aquatic and terrestrial habitats, major sources of anthropogenic noise are associated with transportation networks, which are growing faster than the human population (Barber et al., 2010). According to the World Health Organization (2011), anthropogenic noise is one of the most hazardous forms of pollution that is recognized as a major global pollutant.

In many habitats, noise from natural sources is ubiquitous and it is an important evolutionary selective force (Wiley, 2015). In contrast, anthropogenic noise differs from naturally occurring noise because it is typically loud and low in pitch (Hildebrand, 2009). These different properties make anthropogenic noise a novel stimulus, which can be perceived as an environmental stressor (Kight & Swaddle, 2011; Shannon et al., 2016). Historically, anthropogenic noise has been viewed as a major problem for humans, because it can lead to a wide range of health issues, from simple annoyance to early death, but it can also have negative socio‐economic impacts. Only relatively recently has it been realized that animals also are affected by anthropogenic noise. Understanding the effects of human‐induced environmental changes, such as anthropogenic noise, on animals is crucial because it allows to predict how species will respond to such changes. The degree to which species respond to an external stressor is the sensitivity of a species, with more sensitive species exhibiting larger trait adjustments than less sensitive species (Keinath et al., 2017).

One key trait that allows animals to respond to human‐induced environmental changes is behaviour. A behaviour that is affected by novel environmental conditions is animal communication, which plays a crucial role in many species (Bradbury & Vehrencamp, 2011). Acoustic signals used in communication allow individuals to exchange information about their quality, status, or motivation (Todt & Naguib, 2000). Anthropogenic noise can directly interfere with acoustic signals, and species relying on acoustic signals to communicate might be particularly vulnerable to increasing anthropogenic noise. Over the last decade, an increasing number of studies have investigated the effects of anthropogenic noise on acoustic communication. However, such single studies can neither provide a holistic understanding of the potential effects of noise nor unravel potential species sensitivities to this novel environmental stressor.

We conducted a phylogenetically controlled meta‐analysis to quantify how noise pollution affects acoustic communication and how species differ in their sensitivities to noise. As only carefully controlled experiments allow to establish cause‐and‐effect relationships (Milinski, 1997), we focused on experimental studies to assess the effects of noise without ambiguity. Sufficient data were available to extract 121 effect sizes for six signal components from 23 experimental studies on 31 species. We were able to include data on (a) signal amplitude, two measures of spectral characteristics: (b) minimum frequency, which is the lowest frequency of an acoustic signal; and (c) dominant frequency and peak frequency. Technically, dominant and peak frequencies are not exactly the same as they differ in how they are measured. However, biologically they are comparable as both describe the frequency with the most energy. Therefore, we have grouped dominant and peak frequency in our analysis together. Finally, we extracted data on signal (d) duration, (e) complexity, and (f) rate. For each signal component, we analysed two measures: the magnitude and the direction of signal adjustment. To quantify species sensitivities in response to noise, we calculated heterogeneity, which allows to assess how much of the inconsistencies among effect sizes are attributable to species differences (for details, see Section 2).

We predicted that species will exhibit (a) the Lombard effect, that is, an increase in signal amplitude with increasing anthropogenic noise levels. The diversity of species exhibiting the Lombard effect led to the view that is widespread in vertebrates. Consequently, we expected that inconsistencies among species in amplitude adjustment are low. Other, commonly suggested mechanisms to cope with increasing noise levels are an increase in the frequency of signals and/or signal duration (Brumm & Slabbekoorn, 2005; Patricelli & Blickley, 2006). Therefore, we predicted an increase in (b) minimum frequency, (c) dominant frequency, and (d) signal duration, and that the inconsistencies among species are low. Furthermore, complex habitats are sometimes associated with lower (e) signal complexities (Brown & Handford, 2000; Ey & Fischer, 2009), and consequently, we predicted that signal complexity decreases in noise and that inconsistencies among species are low. Finally, we analysed (f) signal rate and predicted an increase in signalling rate in noise and expected that inconsistencies among species are low. The degree to which species adjust signal components in response to noise and the potential inconsistencies among species will allow us to identify those signal components and species that are particularly sensitive to anthropogenic noise.

## 2 METHODS

### 2.1 Literature search and study selection criteria

We conducted a systematic literature search in Web of Science (on 13 September 2018) and Scopus (on 14 September 2018), searching for studies that reported effects of noise pollution. Our search was limited to peer‐reviewed articles. Our search in Web of Science was carried out on titles, abstracts, author keywords, and keywords plus. In Scopus, our search was carried out on titles, abstracts, keywords, and limited to the document type article. The search was conducted using the following keywords: “anthropogenic noise” OR “noise pollution” OR “environmental noise”, returning 5,921 records in Web of Science and 10,637 in Scopus, respectively (cf. Kunc & Schmidt, 2019). Records were downloaded as BibTeX database files (*.bib) and then merged in R (R Core Team, 2020) using the package REVTOOLS (Westgate, 2018), resulting in a data frame with 16,558 studies. In REVTOOLS, we identified and removed 3,063 duplicates, and for each of the remaining 13,495 studies, we checked the title and abstract to determine whether the research was indeed quantifying the effect of anthropogenic noise pollution (for details see Figure S1). In cases where we could not clarify this from the title and abstract we read the paper to find the relevant information. Additionally, we found 14 eligible studies by checking bibliographies of articles.

To be included in our analysis, the studies had to fulfil the following four criteria (cf. Kunc & Schmidt, 2019): (a) The effect sizes must be obtained from experimental studies. In cases where different amplitudes of noise were played back, we chose the values of those exposures with the highest noise amplitude. (b) The reported details on sample size, measure of central tendency, and measure of spread had to be accessible in the text, figures, or supplementary material. To extract data from figures, we used the software Web plot digitizer (Rohatgi, 2017). (c) The type of stimuli used in noise exposure experiments had to mimic the characteristics of anthropogenic noise, which has most energy in the lower frequencies (Hildebrand, 2009; Kunc et al., 2016; Slabbekoorn et al., 2010). Some noise exposure studies use broadband white noise, which has equal intensity throughout the frequency range, giving it a constant power spectral density. Therefore, this broadband white noise is different from anthropogenic noise, and consequently, studies using broadband white noise exposures could not be included in our analysis. Studies where white noise was low‐pass filtered to mimic anthropogenic noise were included in our analysis, because the characteristics of filtered white noise are similar to anthropogenic noise (Halfwerk & Slabbekoorn, 2009; Lohr et al., 2003). (d) The response to the treatment had to be unambiguously elicited by anthropogenic noise (cf. Kunc & Schmidt, 2019). Overall we obtained 121 effect sizes from 23 experimental studies and 31 species. The details of the studies included in the analysis can be accessed in the data file (Supplement S2).

### 2.2 Choice of effect size and phylogeny

We used the standardized mean effect difference as it is considered a good fit for experimental studies (Nakagawa et al., 2017), specifically, the ‘standardized mean effect difference with heteroscedastic population variances in two groups (SMDH)’ in the R package METAFOR (Bonett, 2008, 2009; Viechtbauer, 2010). To control for phylogeny, we created a phylogenetic matrix of species in the dataset using the Open Tree of Life (Hinchliff et al., 2015). We used the ROTL package (Michonneau et al., 2016) to access the Open Tree of Life in R. ROTL does not calculate branch lengths for trees and thus we calculated these using the compute.brlen function in the APE package (Paradis et al., 2004). A correlation matrix of phylogenetic relatedness among species was then built using APE’s vcv function. This correlation matrix was incorporated in all models in METAFOR so that phylogenetic relatedness among effect sizes could be accounted for as a random effect (Hadfield & Nakagawa, 2010).

### 2.3 Statistical analysis

All statistical analyses were performed in R studio 1.1.463 with R version 3.5.1 (R Core Team, 2020). To account for the non‐independence of effect sizes, we used phylogenetically controlled meta‐analytical multi‐level random‐effects models (Nakagawa & Santos, 2012; Viechtbauer, 2010). Meta‐models were built using the rma.mv function in the package METAFOR (Viechtbauer, 2010). To quantify the magnitude of response to noise, we ran a model on each of the six signal components, including study, effect size, and phylogeny as random factors. As the standardized mean difference approach does not correct for differences in the direction of response variables (Chandler et al., 2019), we applied the folded normal distribution to the mean estimate (cf. Morrissey, 2016a, 2016b, see also Dougherty & Guillette, 2018; Noble et al., 2018). To quantify the direction of response to noise, we ran models on the raw values of each signal component, including study, effect size, and phylogeny as random factors. Circle plots were created with the package CIRCLIZE (Gu et al., 2014). Silhouette drawings are from phylopic.org.

### 2.4 Heterogeneity

Meta‐analysis allows to quantify heterogeneity *I* ^2^_total_, which is the variance that is not due to sampling error or, in other words, the variance in true effects in contrast to the sampling variance (Borenstein et al., 2017). To test whether there was more heterogeneity in effect sizes among studies than could be explained by sampling error alone, we used Cochran's *Q* statistic. This formally tests whether variation in effect sizes is greater among studies than expected if the true effect is identical for all studies (Hedges & Olkin, 2014). However, the ratio assumes a constant within‐study variance, which is not the case as sampling error varies due to studies having different sample sizes (Borenstein et al., 2017); thus, heterogeneity *I* ^2^ should be treated as a measure of ‘inconsistency’ in effect sizes among studies (Borenstein et al., 2017). Therefore, total heterogeneity *I* ^2^ indicates how much of the total variance can be attributed to the total amount of heterogeneity, which is the sum of between‐ and within‐cluster heterogeneity (Konstantopoulos, 2011; Viechtbauer, 2010).

To quantify heterogeneity *I* ^2^ for the multilevel meta‐analytic models, we calculated heterogeneity following (Nakagawa & Santos, 2012). These modified heterogeneity *I* ^2^ partitions the proportion of unknown variance that is not attributable to sampling variance into the contribution of random factors. In our analyses, this is the variance in effect sizes due to phylogenetic relatedness, differences among studies, and differences in within‐study variation. Here, *I* ^2^_effect size_ reflects inconsistencies within studies, *I* ^2^_study_ reflects inconsistencies among studies, *I* ^2^_phylogeny_ are inconsistencies due to phylogenetic relatedness, and *I* ^2^_total_ is the sum of all these values combined. The sum of the percentages of total variation due to these sources equals the traditional *I^2^* (Higgins et al., 2003). High heterogeneity suggests that there may be differences in responses between groups of studies, which can have ecologically important implications (Gurevitch & Hedges, 1999; Hedges & Olkin, 2014).

### 2.5 Publication and time‐lag bias

Publication bias may arise when statistically significant results are more likely to be published than statistically non‐significant results (Borenstein et al., 2009). The resulting bias may lead to unfounded conclusions that can impact the assessment of the factor under investigation (Ioannidis, 1998; Simes, 1987; Stern & Simes, 1997). We checked for publication bias using two widely used approaches: funnel plots and Egger's regression (Egger et al., 1997; Rothstein et al., 2005; Sterne & Egger, 2005). For the Egger's regression, we modified the multi‐level random‐effects models by including the precision of the effect sizes as a moderator (cf. Sanchez‐Tojar et al., 2018). When the intercept of this regression test significantly deviates from zero, the overall relationship between the precision and size of studies included in a dataset is considered asymmetrical, and therefore, biased (Sterne & Egger, 2005). We considered datasets to be biased if the intercept differed from zero at *p* = .1 (Egger et al., 1997). We also tested for a time‐lag bias, that is, that the magnitudes of an effect diminish over time (Jennions & Moller, 2002; Koricheva et al., 2013; Trikalinos & Ioannidis, 2005), which has been reported in different areas of ecology and evolution (Koricheva et al., 2013). To quantify whether significant temporal changes (time‐lag bias) in the magnitude of effects sizes occur in our dataset, we used graphical inspection and a regression (Koricheva et al., 2013).

## 3 RESULTS

Our final dataset included 23 studies reporting data on 31 species. We analysed 121 effect sizes for the difference between control and noise treatment. For each of the six signal components, we quantified both the magnitude and the direction of adjustment of six signal components in response to anthropogenic noise.

### 3.1 Analysis of the adjustments to anthropogenic noise

**FIGURE 1:**
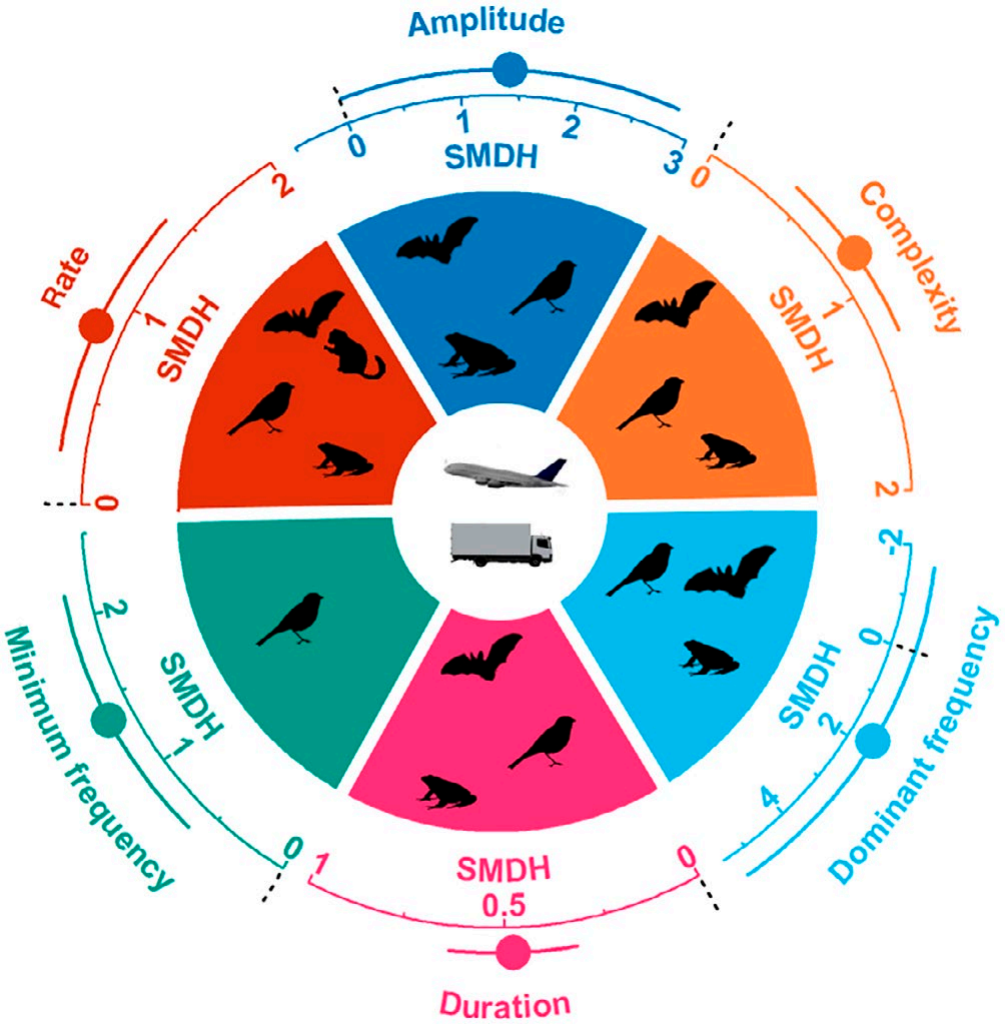
The effects of anthropogenic noise on acoustic communication. Shown is the magnitude of signal component adjustments in standardized mean differences (SMDH) and 95% confidence intervals (CIs) from random effects models. The dashed line at zero indicates no effect of anthropogenic noise and a statistical significant effect of noise occurs if the 95% CI of the SMDH does not overlap zero. For sample sizes of effect sizes, studies and species see Table 1. Silhouettes represent which taxa were included in the analysis of each single component

The majority of signal components deviated significantly from zero in their magnitude of response, and total heterogeneity was high for most components (Figure 1; Table 1a). In contrast to changes in magnitude, species did not show a consistent adjustment in the direction of response as the confidence intervals overlapped with zero for most components. Correspondingly, the total heterogeneity was high for all components and stemmed mostly from inconsistencies among species (Table 1b).

**TABLE 1:** Effect of anthropogenic noise on acoustic signals in animals. (a) Magnitude of adjustments. (b) Direction of signal adjustment. Estimates and 95% confidence intervals (CI) calculated from a phylogenetically controlled meta‐analysis. All effect sizes (ES) are derived from experimental noise exposure studies. Heterogeneity allows to assess how much of the inconsistencies among effect sizes are attributable to phylogenetic relatedness

	Number of			Estimate	*SE*	*z*	CI		*p*	Heterogeneity *I* ^2^ [%]						
	ES	Studies	Species				Lower	Upper		ES	Study	Phylogeny	Total	*Q*	*df*	*p*
(a) Magnitude																
Amplitude	20	8	11	1.42	0.72	1.97	0.01	2.83	.049	0	0	91.78	91.78	89.7	19	<.001
Complexity	7	7	5	0.83	0.21	3.99	0.42	1.24	<.001	43.17	14.09	0	57.25	15.3	6	.018
Dominant frequency	21	8	12	1.84	1.75	1.05	−1.59	5.27	.293	0	0	98.97	98.97	133.4	20	<.001
Duration	28	14	13	0.48	0.08	6.42	0.33	0.63	<.001	0	25.10	0	25.1	22.7	27	.703
Minimum frequency	13	12	10	1.37	0.39	3.50	0.60	2.14	<.001	0	95.28	0	95.28	89.8	12	<.001
Rate	32	14	22	0.85	0.30	2.79	0.25	1.44	.005	0	12.08	68.24	80.31	87.3	31	<.001
(b) Direction																
Amplitude	20	8	11	−0.03	1.29	−0.02	−2.56	2.50	.982	0	0	96.24	96.24	160.7	19	<.001
Complexity	7	7	5	−0.20	0.60	−0.34	−1.38	0.97	.734	15.74	15.6	54.5	85.84	33.8	6	<.001
Dominant frequency	21	8	12	1.61	2.47	0.65	−3.24	6.46	.515	0	0	98.94	98.94	156.6	20	<.001
Duration	28	14	13	−0.14	0.37	−0.39	−0.87	0.58	.698	0	8.16	66.31	74.47	52.3	27	.002
Minimum frequency	13	12	10	1.20	0.48	2.50	0.26	2.15	.013	0	94.12	0	94.12	99.6	12	<.001
Rate	32	14	22	−0.06	0.48	−0.12	−0.99	0.88	.906	0	41.08	48.87	89.95	171.6	31	<.001

**FIGURE 2:**
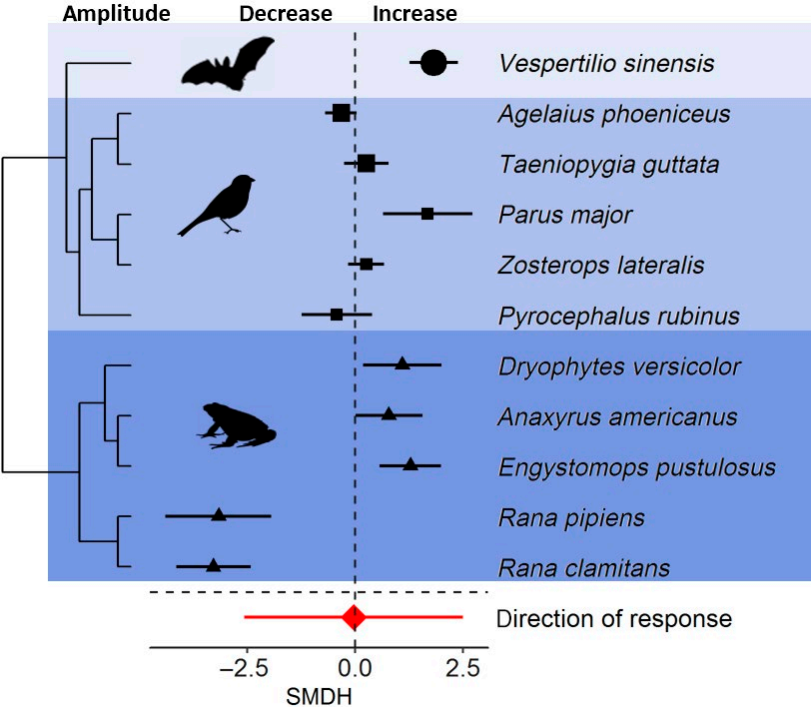
Species sensitivities in signal amplitude in response to anthropogenic noise. Shown are the standardized mean differences (SMDH) and 95% confidence intervals (CIs) from random effects models. The dashed line at zero indicates no effect of anthropogenic noise and a statistical significant effect of noise occurs if the 95% CI of the SMDH does not overlap zero

Signal amplitude changed in response to anthropogenic noise (Figure 1), as some species produced louder signals while other species produced quieter signals (Figure 2). Thus, the magnitude of amplitude adjustments deviated significantly from zero, but inconsistencies among species were high. The high inconsistencies among species suggest that species differ in their magnitude of adjustment to anthropogenic noise. The direction of amplitude adjustments did not deviate significantly from zero, and inconsistencies among species were high, suggesting that the direction of adjustments differs among species (Table 1).

**FIGURE 3:**
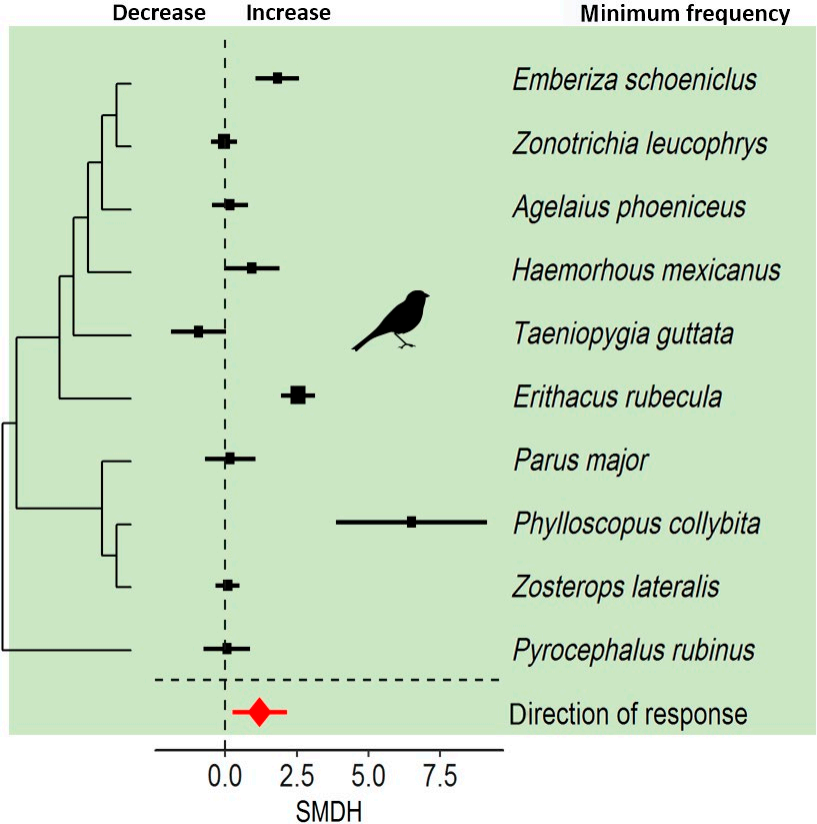
Species sensitivities in minimum frequency in response to anthropogenic noise. Shown are the standardized mean differences (SMDH) and 95% confidence intervals (CIs) from random effects models. The dashed line at zero indicates no effect of anthropogenic noise and a statistical significant effect of noise occurs if the 95% CI of the SMDH does not overlap zero

**FIGURE 4:**
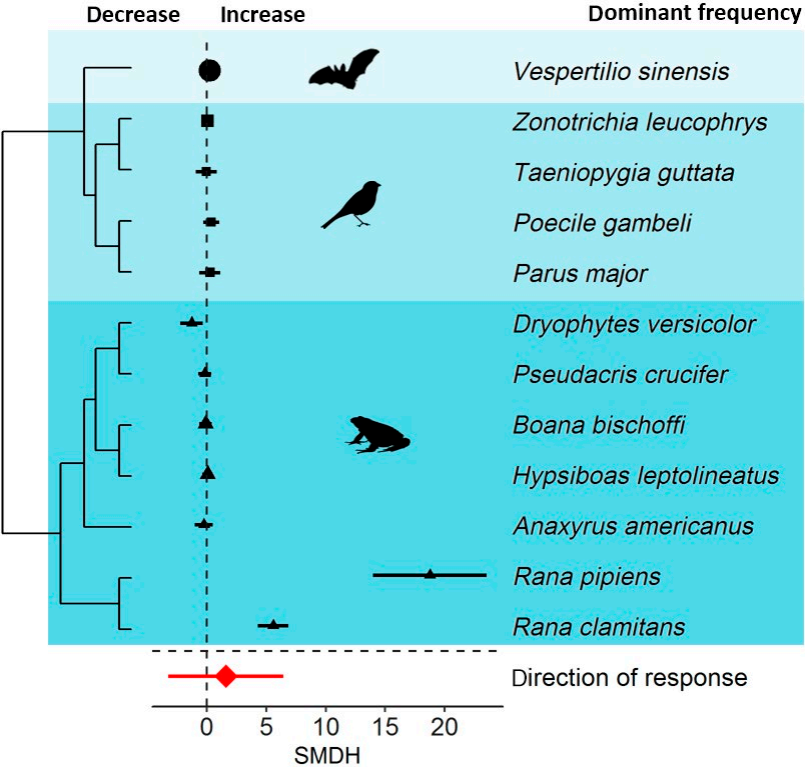
Species sensitivities in dominant frequency in response to anthropogenic noise. Shown are the standardized mean differences (SMDH) and 95% confidence intervals (CIs) from random effects models. The dashed line at zero indicates no effect of anthropogenic noise and a statistical significant effect of noise occurs if the 95% CI of the SMDH does not overlap zero

Animals increased the minimum frequency of their signals in response to noise (Figure 3). Both the magnitude and the direction of minimum frequency deviated significantly from zero. The total heterogeneity was high in both models, but phylogeny did not contribute to the total heterogeneity, suggesting that the magnitude and direction of response did not differ among species (Table 1). Overall, animals did not adjust the dominant frequency of their signals, as both the magnitude and direction of dominant frequency did not deviate significantly from zero. The total heterogeneity was high in both models and stemmed exclusively from inconsistencies among species (Figure 4; Table 1).

**FIGURE 5:**
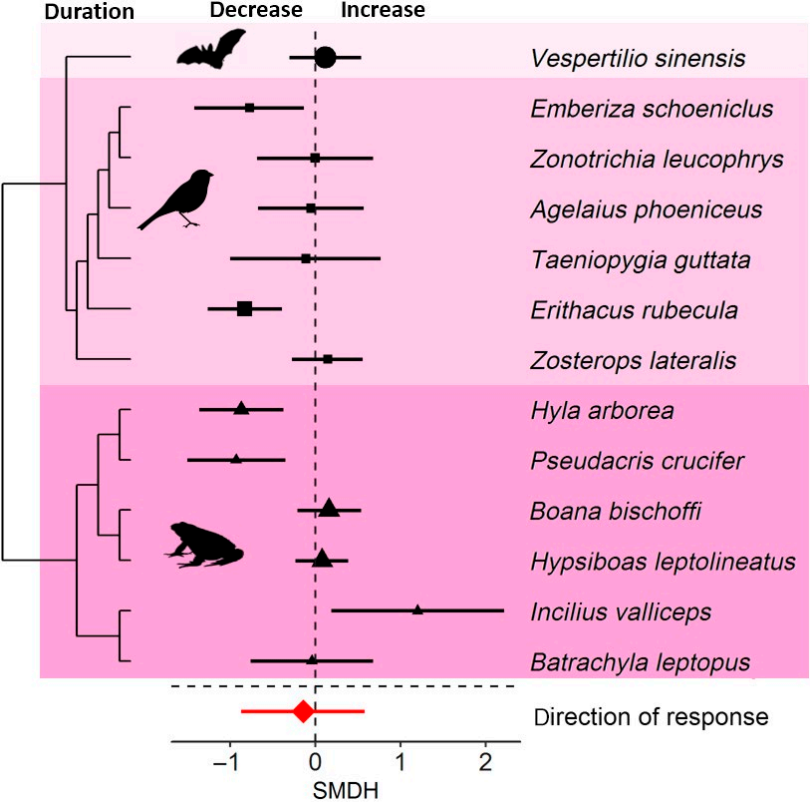
Species sensitivities in signal duration in response to anthropogenic noise. Shown are the standardized mean differences (SMDH) and 95% confidence intervals (CIs) from random effects models. The dashed line at zero indicates no effect of anthropogenic noise and a statistical significant effect of noise occurs if the 95% CI of the SMDH does not overlap zero

Signal duration changed in response to noise (Figure 1) as some species shorten the duration of signals and others increase the duration of signals (Figure 5). Consequently, the magnitude of signal duration deviated significantly from zero in response to noise, but inconsistencies among species were low, suggesting that species did not differ in their magnitude of adjustment. In contrast, direction of change of duration did not significantly deviate from 0, suggesting that species did not adjust signal duration in a consistent pattern (Figure 5). The total heterogeneity for duration was high for the analysis of the direction of response, with inconsistencies stemming mostly from species (Table 1).

**FIGURE 6:**
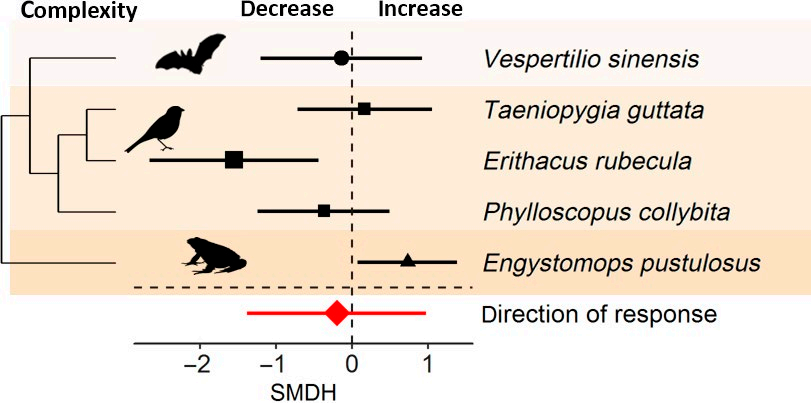
Species sensitivities in signal complexity in response to anthropogenic noise. Shown are the standardized mean differences (SMDH) and 95% confidence intervals (CIs) from random effects models. The dashed line at zero indicates no effect of anthropogenic noise and a statistical significant effect of noise occurs if the 95% CI of the SMDH does not overlap zero

Signal complexity changed in response to noise (Figure 1), as the magnitude of signal complexity deviated significantly from zero. The inconsistencies among species were low, suggesting that the magnitude of signal adjustments did not vary among species. In contrast to the magnitude, the confidence interval of the direction of the effect overlapped with zero and inconsistencies among effect sizes stemmed from all three random effects with inconsistencies among species contributing the most (Figure 6; Table 1).

**FIGURE 7:**
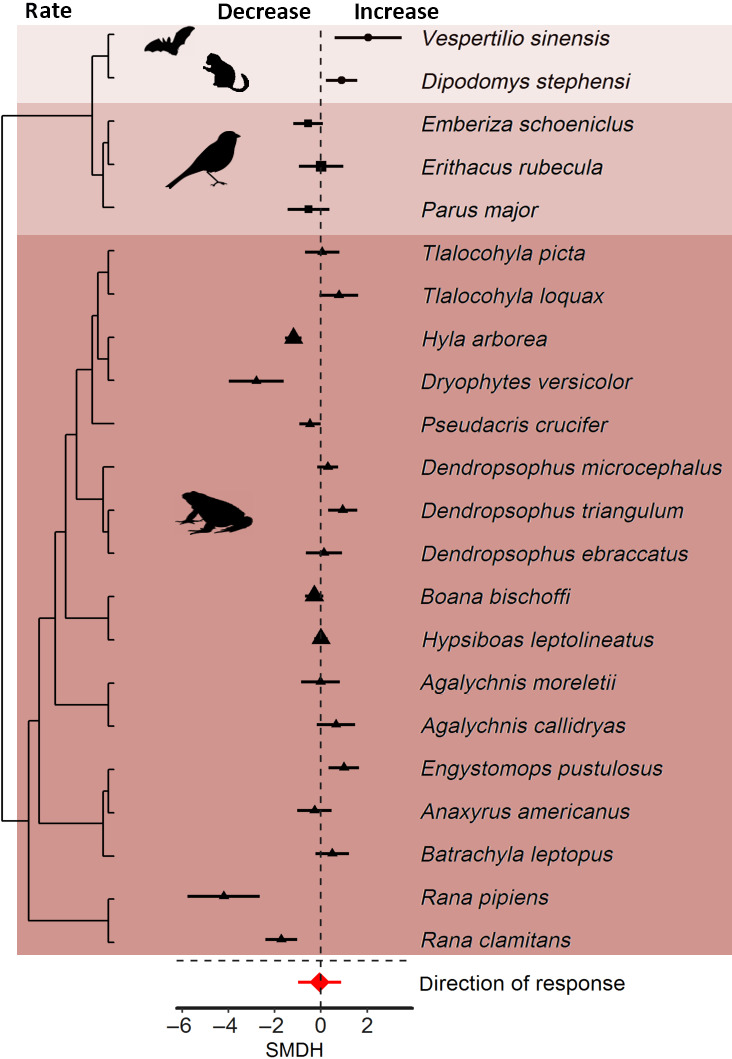
Species sensitivities in signalling rate in response to anthropogenic noise. Shown are the standardized mean differences (SMDH) and 95% confidence intervals (CIs) from random effects models. The dashed line at zero indicates no effect of anthropogenic noise and a statistical significant effect of noise occurs if the 95% CI of the SMDH does not overlap zero

Finally, species changed signal rate in response to noise (Figure 1) as some species decreased and other increased signal rate (Figure 7). The magnitude of signal rate changed significantly in response to noise, but the direction did not. Total heterogeneity was high in both models and inconsistencies among species contributed the most to total heterogeneity (Table 1).

### 3.2 Publication bias and time‐lag bias

Visual inspection of funnel plots for each signal component suggested not much asymmetrical distribution of effect sizes around the meta‐analytic residuals (Figure S2). Egger's regressions indicate some publication bias for some of the six signal components, namely for signal complexity, dominant frequency and minimum frequency (Table S1). Visual inspection of time‐lag plots for each signal component suggested not much influence of year on effect sizes (Figure S3); dominant frequency and minimum frequency showed a decrease in effect sizes with year of publication while signal amplitude showed an increase with year of publication (Table S2).

## 4 DISCUSSION

Animals exposed to anthropogenic noise adjust their acoustic signals, but the direction of adjustments varies among species. This inconsistency in response among species indicates that species differ in their sensitivity to noise. Below, we discuss the results for each signal component separately, highlighting different patterns of response in acoustic components among species, and the potential consequences of these adjustments for each component. Finally, we point out knowledge gaps and challenges that should be addressed in future studies.

### 4.1 Change in signal components

Animals adjusted the magnitude of amplitude to anthropogenic noise (Figure 1); however, the direction of amplitude adjustment among species was highly variable, that is, the inconsistencies among species were high for both the magnitude and the direction of response (Figure 2; Table 1). This is remarkable because an increase in signal amplitude with increasing noise levels (Lombard effect) has been considered as common in birds and mammals (Brumm & Zollinger, 2012). However, our phylogenetically controlled meta‐analysis shows that adjustments in signal amplitude to experimental anthropogenic noise exposure are not as uniform as expected (Figure 2). The study setup of the studies from which we obtained the effect sizes can influence the outcome of the results. For example, in amphibians, there is some disagreement on how widespread the Lombard effect is, because differences in response in closely related species could be explained by study setups (Halfwerk et al., 2016; Love & Bee, 2010; Penna & Hamilton‐West, 2007). Potentially, a meta‐analysis, including moderators and thereby controlling for potential differences in study setups, could help untangling the different factors settling this disagreement. Unfortunately, not many primary studies provide the required data to formally control for such differences in a meta‐analysis.

The majority of the amphibian effect sizes on amplitude derive from one particular study (Cunnington & Fahrig, 2010), which we expect to minimize the effect of observer and study design. In this study, two of the species showed a decrease, and the other two species showed an increase in amplitude, and the study setup among species seem to be the same. While we have to be careful to draw conclusions from such a sample, it allows to rule out some potential explanations for the reported differences among species in general: (a) Differences in how amplitude are measured alone cannot explain the variability among species, as it appears that amplitude was measured in the same way in the four species reported in the study, but we echo the call of Halfwerk et al. (2016) that more carefully calibrated measurements of signal amplitude are needed. (b) The degree of signal overlap with anthropogenic noise alone cannot explain the increase in signal amplitude. It can be argued that only those species whose signals overlap with the spectral characteristics of noise exhibit a response to avoid masking by noise. However, those two amphibian species whose vocalizations overlap with anthropogenic noise, that is, *Rana clamitans* and *Rana pipiens*, decrease signal amplitude in response to noise while the two other species, that is, *Anaxyrus americanus* and *Dryophytes versicolor*, whose vocalizations do not overlap with noise increase signal amplitude (Figure 3 in Cunnington & Fahrig, 2010). Potential explanations for this are that increasing the amplitude may either not pay off when noise levels are so high that even a maximum increase in signal amplitude does not allow effective signal transmission or animals would simply not be able to produce louder signals due to correlations among signal components that allow to increase amplitude (see below). (c) Variation in anthropogenic noise amplitude alone cannot explain the differences in responses among species as the species adjusted the amplitude in different directions. Therefore, if the loudness (amplitude) of anthropogenic noise alone is responsible for signal amplitude adjustments, we did not expect to find these different patterns of response in one study. Thus, our analysis suggests that the most parsimonious explanation for this disagreement is that species differ in their signal amplitude adjustments to increasing anthropogenic noise.

Birds increased minimum frequencies of their songs in response to noise (Figure 1) and responses showed little variation. The inconsistency among species was low for both the magnitude and the direction in response because most species increased minimum frequency (Table 1; Figure 3). An important factor in shaping the evolution of bird song is the habitat with its unique characteristics, for example, in vegetation, topography and ambient noise (Hunter & Krebs, 1979; Morton, 1975; Nottebohm, 1975). For example, pitch components of song, such as minimum frequency and peak frequency, are lower in closed than in open habitats (Boncoraglio & Saino, 2007). Nevertheless, how ambient noise alone shapes acoustic signals is less understood. In little greenbuls, *Eurillas virens*, it seems that differences in minimum frequencies are driven by habitat‐dependent ambient noise characteristics (Slabbekoorn & Smith, 2002). In line with these findings obtained from undisturbed habitats, comparisons between noise‐polluted urban areas and rural areas with less anthropogenic noise suggest that pitch components of signals are frequently adjusted (e.g. blackbirds: Nemeth & Brumm, 2009; great tits: Mockford & Marshall, 2009; Slabbekoorn & den Boer‐Visser, 2006; robins: Montague et al., 2013; reed bunting: Gross et al., 2010). Our finding that minimum frequency is adjusted to increasing noise levels is in line with both observational and experimental studies (cf. Roca et al., 2016). It should be noted that the way how minimum frequency is measured is debated, and we support the call of Brumm and Bee (2016) to be more careful on how to measure minimum frequency changes. More importantly, from a biological point of view, little is known what kind of information value minimum frequency actually provides.

Dominant frequency did not change significantly in response to anthropogenic noise, but inconsistencies among species were high (Table 1). These inconsistencies among species can be explained by two amphibian species (*R. clamitans*, *R. pipiens*) responding strongly with an increase in dominant frequency (Figure 4). Why are these two species responding so strongly? Methodological differences alone can be ruled out (see above). However, the dominant frequency of calls of these two species overlaps with anthropogenic noise. Therefore, these species might try to mitigate the masking effects of anthropogenic noise by increasing the pitch of their acoustic signals (cf. Brumm & Slabbekoorn, 2005), which is in line with adjustments of minimum frequency in birds discussed above. In amphibians, adjustments in dominant frequency can affect agonistic interactions between males. In some anuran species, information encoded in frequency components is important, because dominant frequency can be negatively correlated with body size (Gerhardt & Huber, 2002), allowing individuals to assess each others’ fighting ability (Davies & Halliday, 1978). In green frogs (*R. clamitans),* males adjust dominant frequency in response to an opponent's calls, suggesting that males use information encoded in dominant frequency to assess the size of an opponent during aggressive encounters (Bee et al., 1999). Taken together, changes in dominant frequency can provide accurate information on how males respond to other males (Burmeister et al., 2002). Therefore, anthropogenic noise could limit the correct assessment and change the outcomes of fights as larger individuals producing low‐frequency calls may increase the frequency of calls in response to noise, which would prevent other individuals from assessing the size and thus fighting ability of males correctly.

Animals adjusted the magnitude of duration in response to noise, and inconsistencies was low, indicating that the magnitude did vary little among species (Figure 1; Table 1). However, the direction of adjustments was variable among species (Figure 4). In many species, signal duration can contain information about the sender and signal duration can be important in reproductive decisions. For example, female insects and anurans often prefer long signals over short signals (Gerhardt & Huber, 2002). In birds, male blue tits singing longer songs had a higher extra‐pair reproductive success than males singing shorter songs (Kempenaers et al., 1997). Preferences for longer signals in females may be relative rather than absolute, and thus differences among signalling males would be preserved, allowing females to make a decisions based on this signal component. However, if signal duration has to be reduced to a new optimum in noise, individuals producing shorter signals might not have to adjust duration as much as individuals producing longer signals. Thus, changes in signal duration in response to noise can potentially affect reproductive success.

The magnitude of signal complexity changed significantly in response to noise (Figure 1); however, the direction of adjustment did not show a consistent significant adjustment (Figure 6). Signal complexity is important in many contexts of animal communication. For example, in sexual selection where females prefer males producing more complex songs and more complex songs can also be more effective in territory defence (summarized in Catchpole & Slater, 2008). Consequently, such reduction in signal complexity in response to noise can negatively affect territory defence and/or reproductive success. This would also explain observational findings that reproductive success of male birds in noise polluted territories is lower than that in less polluted territories (great tits: Halfwerk et al., 2011; ovenbirds: Habib et al., 2007; reed bunting: Gross et al., 2010). Notably, one anuran species, *Engystomops pustulosus*, increased signal complexity in response to low‐frequency noise but the reason for this is unknown. Data on signal complexity in the context of anthropogenic noise are relatively sparse. Thus, more standardized noise exposure studies are needed to get a holistic understanding of changes in signal complexity and their consequences in response to anthropogenic noise.

Animals adjusted signal rate in response to noise (Figure 1), but the direction of adjustment among species is highly variable. The variable response in signal rate is reflected in inconsistencies among species contributing most to total heterogeneity in both analyses (Table 1). Thus, signal rate does not only differ in magnitude but also in the direction of response among species. Experiments in many species of insects and anurans show that females prefer signals produced at high rates over the same signals produced at low rates (Gerhardt & Huber, 2002). Similar patterns have also been reported in bird interactions where higher signal rates are more effective than lower rates (Catchpole & Slater, 2008). Given the inconsistencies in responses among species in signalling rate (Figure 7), it seems that some species are more prone to adjustments than others. Notably, not only acoustic signals produced vocally can be affected by noise: the Stephen's kangaroo rat *Dipodomys stephensi* adjusted signal rate by increasing foot drumming (Shier et al., 2012).

To summarize, our analysis shows that animals adjust their signals to anthropogenic noise by changing individual signal components, which is reflected in the magnitude of responses deviating from zero in most cases. However, the direction of response varies among species, which is reflected by the overlap with zero, but also in the inconsistencies among species. Meta‐analyses in ecology and evolution often include effect sizes that were collected in different ways (e.g. observations vs. experiments). Here, we only included effect sizes from experimental studies, because only experiments allow to establish cause‐and‐effect relationships (Milinski, 1997), demonstrating that the adjustments to anthropogenic noise are indeed based on species sensitivities.

### 4.2 Publication bias and time‐lag bias

We found some indication that studies reporting large adjustments in some signal components were over‐represented in our dataset, namely, signal complexity and pitch components (see Table S1). This pattern could reflect publication bias and selective reporting, whereby significant findings are more likely to be published and reported than non‐significant results (Jennions et al., 2013). A potential publication bias in these signal components is not surprising, because signal complexity is widely studied in different taxonomic groups using acoustic signals in various contexts (e.g. Catchpole & Slater, 2008; Gerhardt & Huber, 2002). Slabbekoorn and Peet (2003) were the first to report a relationship between pitch and anthropogenic noise. This study certainly inspired many authors to focus on the relationship between pitch and noise, which may explain the over‐representation of larger differences in our dataset. To avoid publication bias in the future, we consider it of utmost importance that all signal components that are measured in an acoustic analysis are reported, and not only those that turned out to be statistically significant. We also found some evidence of time‐lag bias for dominant frequency, minimum frequency, and signal rate (see Table S2). The decrease in effect size of dominant and minimum frequency can be explained with one study reporting a very large effect size early on (Figure S3). In contrast to the definition of time‐lag bias, that is, a decrease in effect size over time, signal rate effect sizes increased.

### 4.3 Short‐term adjustments or micro‐evolutionary response?

Adjustments to changes in the environment can occur through either microevolutionary responses to natural selection or phenotypic plasticity (West‐Eberhard, 2003). Phenotypic plasticity allows individuals to adjust immediately to changes in the environment. Until now, most of the phenotypic changes observed in response to other human‐induced environmental changes are found to be based on phenotypic plasticity (Hendry et al., 2008). The effect sizes in this and a previous meta‐analysis (Kunc & Schmidt, 2019) are obtained from short‐term noise exposure experiments, demonstrating that phenotypic plasticity is a key mechanism for animals to adjust to human‐induced environmental changes. While Kunc and Schmidt (2019) showed that adjustments in response to noise in anatomy, physiology, and morphology can be explained by phenotypic plasticity, our analyses show that behavioural plasticity can explain immediate adjustments in signalling.

### 4.4 Future directions

What do our results mean for future studies investigating the effects of human‐induced environmental changes, and what knowledge gaps have to be filled? The inconsistencies among species in their response to a novel environmental stressor highlight the need to focus on disentangling the mechanisms causing differences in species sensitivities. This is important because species sensitivities have direct implications for conservation, which only can be successful if they are tailored to the requirements of the target species (Caro, 2007).

Currently, we have little understanding of how adjustments of single signal components affect individuals on both the proximate and ultimate level, and how these translate into populations and ecosystems. To investigate such transitions, we have to keep at least two things in mind: (a) Species sensitivity only describes the degree to which species respond to changes (Keinath et al., 2017), and adjustments to noise do not have to be negative per se (Kunc & Schmidt, 2019). Our analysis shows that noise has an effect on acoustic signals, but whether an effect may be negative or positive in a biological sense may depend on the species or a given context (Kunc & Schmidt, 2019). For example, producing louder signals may enhance communication efficiency in noisy environments, but producing louder signals may also attract more predators. (b) The functional importance of trait components predicting species sensitivities to noise: Acoustic signals are the traits often shown to be sexually selected and thus directly determining the reproductive success (Andersson, 1994). For example, in some species, females prefer males that produce more complex signals, which are often also more effective in male–male interactions (Catchpole & Slater, 2008). Thus, adjustments in one component, such as a decrease in signal complexity, can have a direct negative impact on the reproductive success of an individual. However, it is rarely the case that both the functional importance and the effect of noise on a specific component are established for a species. Thus, to understand how human‐induced environmental changes affect the overall expression of traits, we have to consider relationships among components (Price et al., 2003).

Phenotypic integration provides a framework to disentangle the relationships among components of complex traits (Pigliucci, 2003). Phenotypic integration has been successfully used to demonstrate that anthropogenic noise hampers the overall expression of bird song (Montague et al., 2013). Therefore, future studies should not only quantify adjustments of single components of acoustic signals in isolation but also consider changing relationships among components in response to noise. This would also resolve the ongoing discussion of whether or not the increase in pitch is only a by‐product of the Lombard effect (although recent research in great tits suggests that these two components are adjusted independently; Zollinger et al., 2017). This is in line with our cross‐species comparison indicating that frequency and amplitude are not correlated per se as these two parameters show no consistent direction in response to noise in those species where changes in frequency and amplitude were measured; specifically in *R. clamitans* and *R. pipiens* where dominant frequency increased while amplitude and call rate decreased (Figures 2, 4 and 7). This is notable for several reasons: (a) the calls of these two amphibian species overlap with anthropogenic noise and thus we would expect an increase in amplitude and (b) that correlations among signal components might be species specific as some species increase amplitude and pitch while others show an opposite pattern, and (c) as all effect sizes stemmed from the same study inconsistencies among studies are unlikely (see above). Thus, underlying correlations among trait components could explain adjustments of acoustic signals in response to noise. For example, while species adjusted the magnitude of duration, we did not find a consistent pattern in the direction. One explanation for this could be correlation among signal duration and signal rate. If one of the first responses to increasing background noise levels is an increase in signal redundancy, that is, signal rate, then animals might have to trade‐off this with signal duration; in other words, to increase signal rate, animals might have to reduce signal duration. Consequently, analysing trait components within the integration framework would allow to explore whether underlying correlations among trait components can explain component‐specific adjustments in noise (Montague et al., 2013). Understanding the drivers of species‐specific component correlations would allow to unravel the underlying mechanisms of signal production, which can help to explain differences in species sensitivities.

To unravel how anthropogenic noise affects the entire communication process, we need to incorporate not only the sender producing the signal but also potential consequences of noise for the receiver. There is a reasonable amount of experimental studies available to develop a holistic understanding of the effects of anthropogenic noise for the sender, but only a few studies have looked at the receiver side. These few studies suggest that anthropogenic noise also affects the receiver (e.g. female response latencies: Bee & Swanson, 2007; mate location: Bent et al., 2018; Schmidt et al., 2014; perception: Pohl et al., 2012; Vasconcelos et al., 2007; territory defence: Mockford & Marshall, 2009; and vocal interactions: McMullen et al., 2014). The small number of effect sizes available for receiver responses does not allow to conduct formal meta‐analyses yet.

Furthermore, signal adjustments may not only have direct impact on the individual level but could also affect higher processes. For example, signal adjustment in response to noise can affect sexual selection processes on two non‐mutually exclusive levels: First, within species, noise might lead to the homogenization of traits in a population which may decrease the opportunities for potential receivers to discriminate reliably among senders. For example, males that are able to produce more complex songs resort to less complex songs in a noisy environment to get the message across (McLaughlin & Kunc, 2013). Consequently, receivers cannot discriminate any longer between senders that have the potential to produce complex songs and those that have not. Thus, if signal complexity is a reliable signal for female choice under natural conditions, anthropogenic noise erodes this sexual selection process. Second, on the species level, species with more complex signals might suffer more from increasing noise pollution than species using simpler songs, which may translate in different abundances among noise polluted habitats and pristine habitats.

Finally, in our analysis, we included effect sizes from studies that experimentally exposed animals to noise and compared an animal's response to a baseline level, which is often the natural background noise levels in an individual's habitat. These studies allow quantifying the direction and the magnitude of adjustment in response to noise. However, there is an important thing to keep in mind: these studies do not allow to rule out that animals just respond to any change in the acoustic environment. This is important if the severity of impact of noise has to be assessed. Only if the response to anthropogenic noise and a control treatment differs, we can safely assess the effect of anthropogenic noise. To date, only a few studies have carried out such an approach, demonstrating that adjustments to anthropogenic noise differ from adjustments to other acoustic changes (e.g. Gross et al., 2010; Kunc et al., 2014; Shier et al., 2012).

## 5 CONCLUSIONS

Animals experimentally exposed to anthropogenic noise adjusted their acoustic signals. Given the importance of communication across the animal kingdom, noise has the potential to affect social relationships among individuals in many species. We found different patterns of response among species within trait components, which highlights the need to disentangle the underlying mechanisms why species differ in their sensitivity to human‐induced environmental changes. The difference in response among species has important implications for legislative bodies to enable effective conservation: it is simply not enough to assess the consequences of environmental stressors such as noise based on a few species because a ‘one size fits all legislation’ does not guarantee to protect species effectively due to differences in species sensitivities. Conservation is traditionally concerned with preserving biodiversity and the habitats that organisms are dependent upon. Given the effects of noise on animals across taxa, natural soundscapes to which species have adapted to are crucial to ensure effective conservation. The value of pristine soundscapes has been acknowledged by regulating bodies which have begun to consider protecting natural soundscapes (e.g. Dumyahn & Pijanowski, 2011; Pijanowski et al., 2011). There is no doubt that tackling human‐induced environmental changes, such as noise pollution, is a crucial societal and economic challenge that will ultimately determine the health of both ecosystems and organisms, including humans.

## Supporting information

Supplementary Material

Supplementary Material

Supplementary Material

Supplementary Material

## Data Availability

The data that support the findings of this study are available in the supplementary material of this article.
