# Supplementary material for: Species sensitivities to a global pollutant: A meta‐analysis on acoustic signals in response to anthropogenic noise
Source: Glob Chang Biol. 2020 Dec 1;27(3):675–88. doi: 10.1111/gcb.15428 (PMC7839775; doi:10.1111/gcb.15428)
Supplement: Supplementary file 1 — Supplementary Material [file GCB-27-675-s001.pdf]

Supplement S1

Supplemental Material for
Species-specific sensitivities to a global pollutant: a meta-analysis on acoustic signals in response to anthropogenic noise

Hansjoerg P. Kunc and Rouven Schmidt

Email: kunc@gmx.at

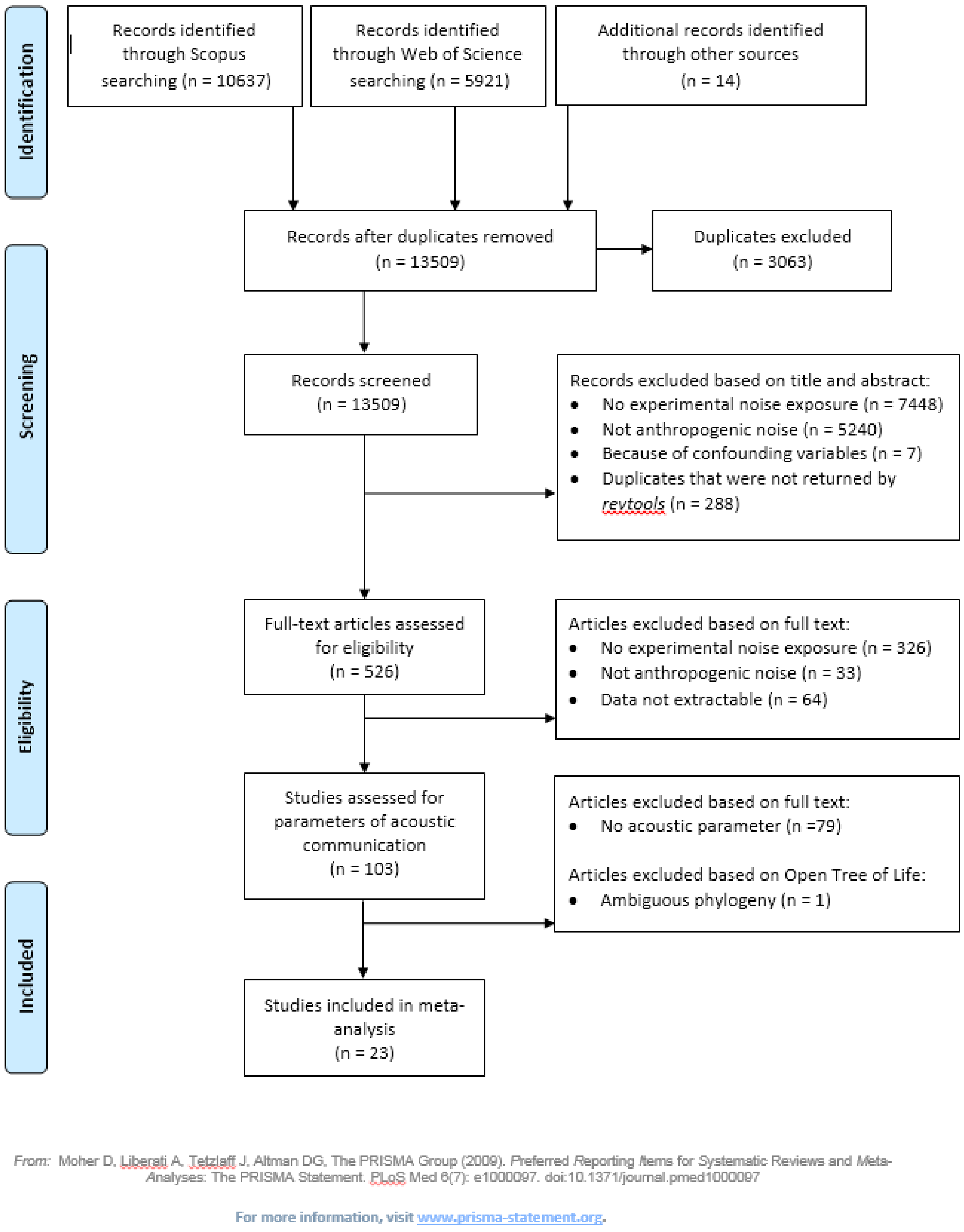

F I G U R E S1 PRISMA flow diagram of the different steps involved in data collection and data selection (following Moher et al., 2009).

TABLE S1 Quantification of potential publication bias for each of the six signal components using intercept estimates from Egger's regression.

	sample.size	studies	species	mean	se	z	lower CI	upper CI	p
amplitude	20	8	11	0.84	1.51	0.56	-2.11	3.8	0.5770
complexity	7	7	5	-2.75	1.46	-1.89	-5.6	0.11	0.0591
dominant.frequency	21	8	12	6.65	3.84	1.73	-0.87	14.17	0.0829
duration	28	14	13	-0.05	0.56	-0.1	-1.15	1.05	0.9235
minimum.frequency	13	12	10	3.44	1.48	2.32	0.53	6.35	0.0205
rate	32	14	22	-0.86	0.76	-1.14	-2.35	0.62	0.2551

TABLE S2 Quantification of time-lag bias for each of the six signal components on mean differences.

	sample.size	studies	species	mean	se	z	lower CI	upper CI	p
amplitude	20	8	11	0.26	0.14	1.91	-0.01	0.53	0.0562
complexity	7	7	5	0.14	0.15	0.93	-0.16	0.44	0.3530
dominant.frequency	21	8	12	-0.62	0.38	-1.63	-1.36	0.12	0.1026
duration	28	14	13	0.06	0.04	1.58	-0.02	0.14	0.1152
minimum.frequency	13	12	10	-0.31	0.15	-2.05	-0.61	-0.01	0.0399
rate	32	14	22	0.15	0.08	1.75	-0.02	0.31	0.0801

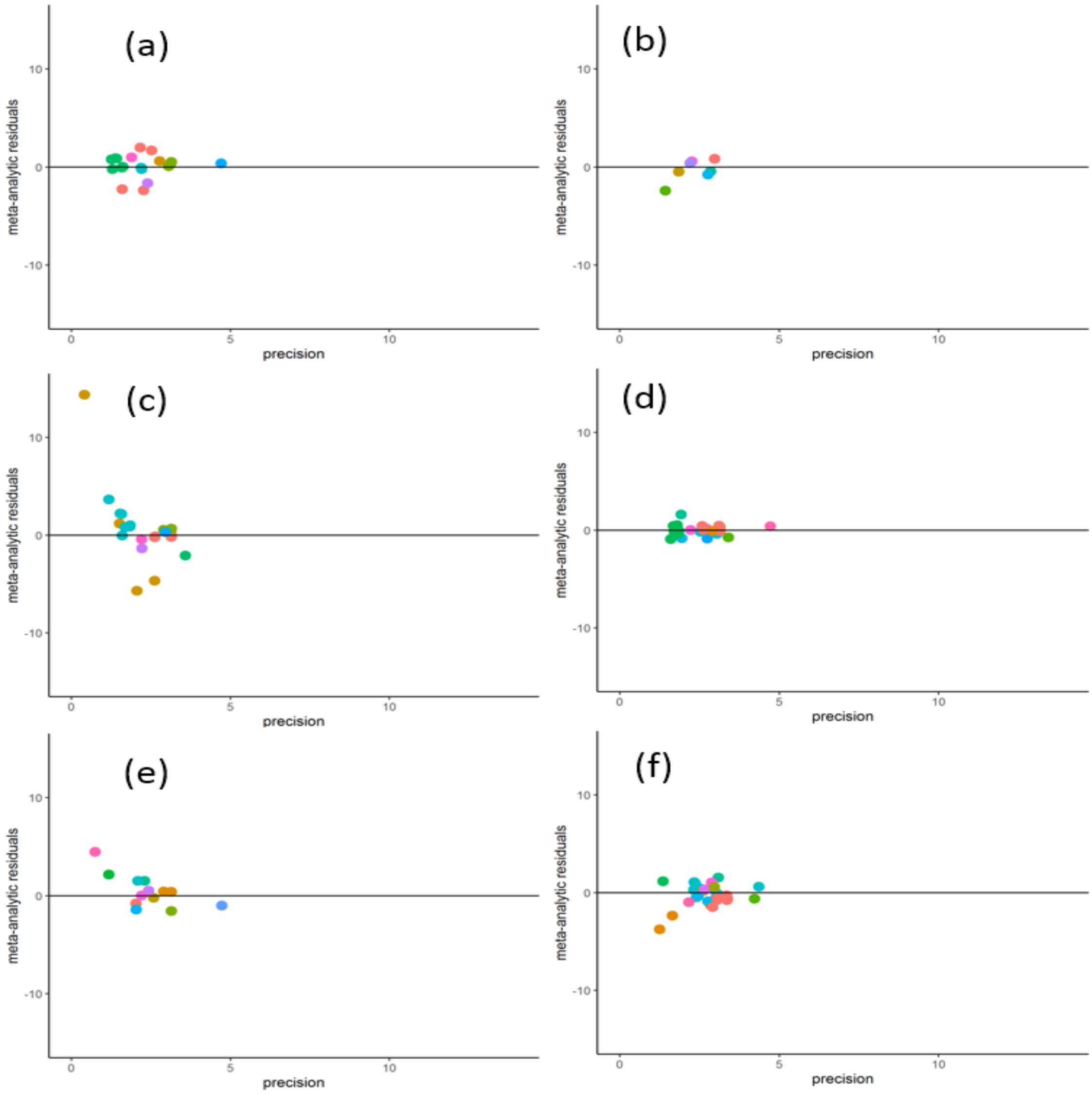

F I G U R E S2 Funnel plots for each of the six signal components of the meta-analytic residuals against their precision for the meta-analysis: (a) amplitude (b) complexity (c) dominant frequency (d) duration (e) minimum frequency (f) signal rate. Each point represents an effect size and each colour represents a study (for sample sizes see Table 1).

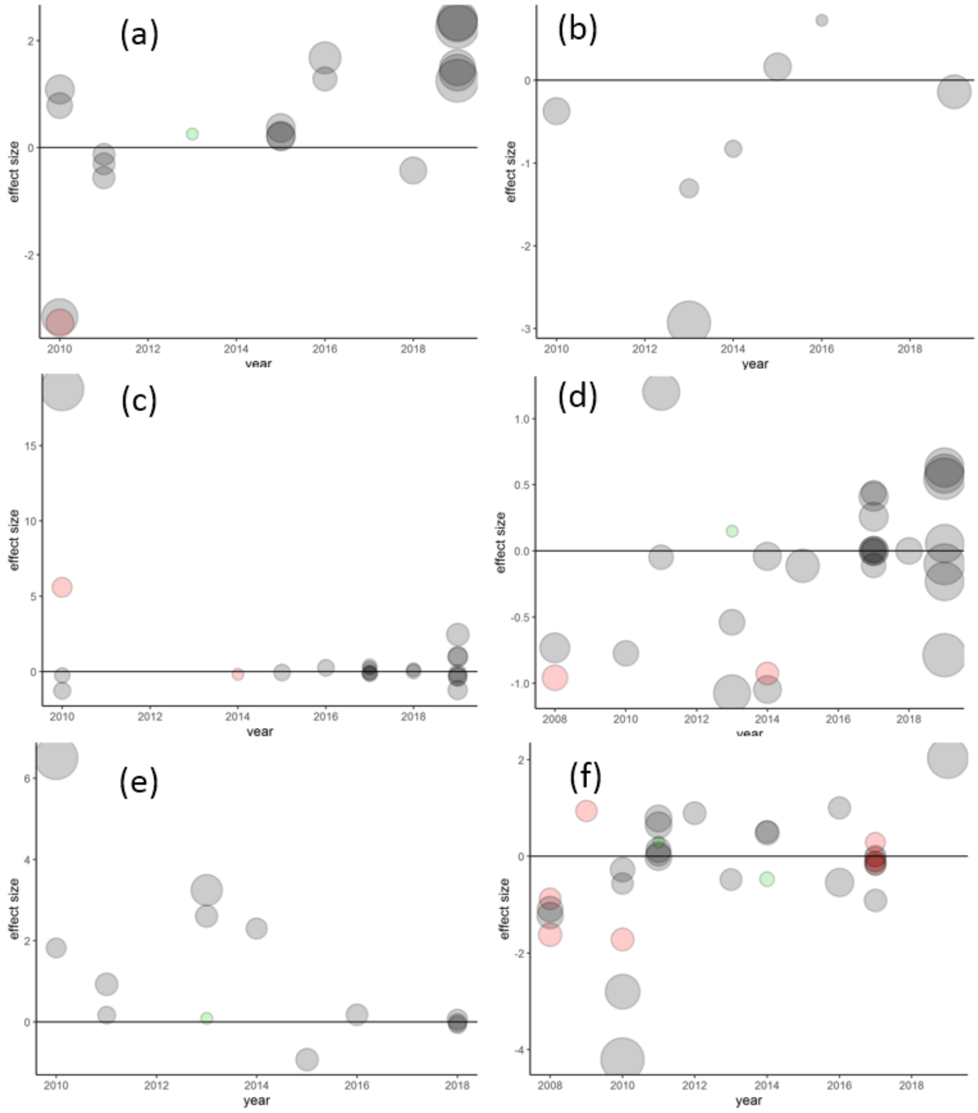

F I G U R E S3 Time-lag bias of the six signal components. Estimates are shown as the standardized mean differences: (a) amplitude (b) complexity (c) dominant frequency (d) duration (e) minimum frequency (f) signal rate. Each point represents an effect size and each colour represents a study (for sample sizes see Table 2). The size of the circle represents the sample variance. The colour represents the sample size for each effect size (colours are grouped into following categories: grey: <= 20, red: 21- 30: green > 30 sample size for each effect size).

References

Moher, D., Liberati, A., Tetzlaff, J., Altman, D. G., & Grp, P. (2009). Preferred Reporting Items for Systematic Reviews and Meta-Analyses: The PRISMA Statement. *Journal of Clinical Epidemiology*, *62*(10), 1006–1012. https://doi.org/10.1016/j.jclinepi.2009.06.005
